# Impulsivity Mediates Associations Between Problematic Internet Use, Anxiety, and Depressive Symptoms in Students: A Cross-Sectional COVID-19 Study

**DOI:** 10.3389/fpsyt.2021.634464

**Published:** 2021-01-28

**Authors:** Julija Gecaite-Stonciene, Ausra Saudargiene, Aiste Pranckeviciene, Vilma Liaugaudaite, Inga Griskova-Bulanova, Dovile Simkute, Rima Naginiene, Laurynas Linas Dainauskas, Gintare Ceidaite, Julius Burkauskas

**Affiliations:** ^1^Laboratory of Behavioral Medicine, Neuroscience Institute, Lithuanian University of Health Sciences, Palanga-Kaunas, Lithuania; ^2^Laboratory of Biophysics and Bioinformatics, Neuroscience Institute, Lithuanian University of Health Sciences, Kaunas, Lithuania; ^3^Department of Informatics, Vytautas Magnus University, Kaunas, Lithuania; ^4^Department of Neurobiology and Biophysics, Institute of Biosciences, Vilnius University, Vilnius, Lithuania; ^5^Laboratory of Toxicology, Neuroscience Institute, Lithuanian University of Health Sciences, Kaunas, Lithuania

**Keywords:** Problematic Internet Use, anxiety, depression, impulsivity, COVID-19

## Abstract

**Background:** Problematic internet use (PIU) is a serious global mental health issue that especially manifested during the Coronavirus disease (COVID-19) pandemic. Engagement in PIU as an impulsive coping with mental distress may pose a long-lasting threat to develop anxiety and depressive disorders. The first aim of our study was to investigate the prevalence of PIU and mental distress symptoms during the COVID-19 pandemic among university students in Lithuania. The second aim was to test the hypothesis that PIU affects anxiety and depressive symptoms through the mediating role of impulsivity.

**Methods:** The cross-sectional study was comprised of 619 university students (92.9% females and 7.1% males) with a mean age of 22 ± 3 years who participated in an online survey from May to November, 2020. Participants completed the following scales: the Problematic Internet Use Questionnaire-9, the Generalized Anxiety Disorder Questionnaire-7, the Patient Health Questionnaire-9, and the Barratt Impulsiveness Scale-11. K-means cluster analysis and one-way multivariate analysis of variance were used for group comparison in terms of internet use time and habit change during COVID-19 pandemic. Structural equation modeling was applied to examine the mediating effect of impulsivity in association between PIU and mental distress, while controlling for age.

**Results:** In sum, 45.1% of the participants reported PIU and 38.1% had markedly expressed symptoms of anxiety while 43.6% of the students reported moderate to severe depressive symptoms. During the COVID-19 pandemic 76% of the students reported at least moderate increase in their internet use time. Anxiety and depressive symptoms were significantly higher in the group of frequent internet users. The results of the structural equational modeling analysis showed a statistically significant effect of PIU on subjective anxiety symptoms and the statistically significant effect of PIU on subjective depression symptoms, both mediated via impulsivity.

**Conclusions:** During COVID-19 pandemic, PIU, anxiety and depression symptoms are highly prevalent among students. Findings also suggest that relationships between PIU, anxiety and depressive symptoms are mediated via impulsivity. These results underscore the importance of the inclusion of impulsivity factor in the studies analyzing longitudinal effects of PIU on mental distress during COVID-19 pandemic.

## Introduction

The first research on the problematic internet use (PIU) emerged two decades ago in the UK and the USA (1, 2). Since then, research has enabled the field to advance considerably, resulting in clinicians and researchers recognizing PIU across different online activities (3). PIU is now considered to comprise a diverse group of complex behaviors, ranging from excessive gambling, online shopping, cybersex and prolonged viewing of pornographic content, to exceedingly frequent email checking, social media use and cyberbullying (4, 5), all of which can cause significant impairment of everyday functioning in some individuals. In fact, PIU has an estimated prevalence reaching up to 27% among citizens and across nations (4, 6) with an increased risk for children and young people (7–9).

Students may be particularly vulnerable to internet addiction, as they have largely unfettered, unsupervised access to the internet and are responsible for their own time management. Several meta-analyses and multi-center studies suggest that prevalence rates of PIU among students might be even higher than in the general population and may range from 27.0 to 30.1% (10, 11). The recent review that examined students in Southeast Asia has also showed the prevalence of PIU to range from zero to 47.4%, resulting in significant impairment manifested as insomnia, daytime sleepiness and eye strain (12). Also, most up to date studies, performed in student populations, suggest PIU to be associated with academic procrastination (13), poor quality of life (14, 15), severe psychiatric disorders (16–18), and even suicide attempts (19). PIU, as an addictive behavioral pattern, is also found to be comorbid with other addictive disorders, such as substance abuse among youth, including cannabis and alcohol use (20) as well as gambling disorder (21, 22).

Recent guidelines on coping with mental distress caused by the Coronavirus disease (COVID-19) pandemic suggest that PIU poses a threat to develop anxiety and depressive disorders (23). However, studies also suggest that several psychiatric disorders, including depression and anxiety disorder, are conditions that may act as predisposing factors for the development and maintenance of PIU. Similarly, mental distress (i.e., anxiety and depressive symptoms) has been shown to be as a possible perpetuating factor that predicted increased levels of PIU (24, 25). This notion was partially confirmed in a longitudinal study by Wartberg et al. (26) showing that current PIU symptomatology was predicted by stronger emotional distress measured at baseline (26). However, another longitudinal study performed in a large sample of Australian adolescents (*N* = 2,809) showed that particularly compulsive PIU leads to emotional problems, such as difficulties pursuing goals in the presence of distress (27). Thus, in terms of causal relationship, the role of mental distress can be viewed as both the predisposing factor as well as the perpetuating/maintaining factor in the development and severity of PIU. Since the frequency of and the dependence on internet use has increased during COVID-19 pandemic (28), it is of crucial importance to pay a particular attention to PIU in order to understand the interplay between PIU and mental health problems that it may pose.

The role of impulsivity in the relationship between PIU, anxiety and depressive symptoms is still under debate (29). A study by Yücens and Üzer (30) analyzed factors related to PIU in a sample of 392 medical students in Turkey, suggesting that mental distress factors rather than impulsivity play a cardinal role in PIU (30). However, the study by Zhang (31) comprising 459 undergraduate students in China found that impulsivity in particular mediated the relationship between PIU and neuroticism (31). A recent Italian study involving 244 university students found that PIU was associated with high attentional impulsivity and depressive symptoms (32). The same relationships were observed in the study analyzing data of 1,600 Indian college students which provided evidence of associations between PIU symptoms of depression, anxiety and impulsivity (33). A study by Wang et al. (34) comprising 4,313 students showed that behavioral characteristics such as effort control and impulsivity might be related to the severity of PIU (34). On the other hand, another study analyzing a community sample of 15,023 individuals reported that personality characteristics better explain PIU rather than the impulsivity itself (35). However, in this particular study participants' depression and anxiety levels were not evaluated.

As indicated by aforementioned works, the interplay between PIU and mental distress (i.e., anxiety and depressive symptoms) in relation to impulsivity is an important relationship to investigate, as it would inform clinicians on the mechanism of the disordered behavior formation. Thus, the first aim of our study was to investigate the prevalence of PIU and symptoms of mental distress during COVID-19 pandemic among university students in Lithuania. The second aim was to test the hypothesis that PIU affects anxiety and depressive symptoms through the mediating role of impulsivity.

## Materials and Methods

### Study Procedure

Students from three major universities in Lithuania were invited to participate in an anonymous online survey during May and November, 2020. The invitation was sent through social media, university websites and the e-mail. Participants completed scales measuring PIU (the Problematic Internet Use Questionnaire, PIUQ-9), anxiety (the Generalized Anxiety Disorder Questionnaire, GAD-7), depressive symptoms (the Patient Health Questionnaire, module for depressive symptoms, PHQ-9), and impulsivity (the Barratt Impulsiveness Scale, BIS-11). Relevant socio-demographic characteristics, additional questions related to changes in internet use frequency and habits (in a five point Likert scale, where “zero” represents no change, and “five” represents extreme changes) during COVID-19 pandemic were also included. The study received the approval from the Bioethics committee and conformed to the principles outlined in the Declaration of Helsinki.

A website was created containing an introduction to the study and questionnaires. A website and data of the answers were hosted on secured servers of Lithuanian University of Health Sciences. To ensure participant's anonymity, no questions were given that would compromise their identity. The website and its design was lightweight and minimalistic, comprising one page with tabulations for separate scales, to make it easy to access, navigate and use. An online consent was provided for each participant for agreement before starting the survey. No incentives were given upon completion.

### Measures

PIU was evaluated employing the nine-item PIUQ-9 questionnaire (36). The PIUQ-9 is a short self-report instrument, which measures three aspects of PIU – an obsession, a neglect, and a control disorder. Nine-scale items are evaluated using a five-point Likert scale, ranging from “Never” to “Always/Almost always.” Total scores range from 9 to 45, with higher scores indicating higher risk of PIU. The previous studies demonstrated appropriate psychometric properties of the PIUQ-9 across a number of European languages and cultures (36, 37). Based on the previous study in a sample of Lithuanian students, a cut-off value of >20 was used for screening markedly expressed PIU symptoms. In the present study, the PIUQ-9 also demonstrated good internal consistency, Cronbach's alpha was 0.84.

The PHQ-9 (38) is a brief self-report tool for screening, diagnosing, monitoring and measuring the severity of depression. Nine items of the questionnaire are based on the depression diagnostic criteria of Diagnostic Statistical Manual-IV; possible response options range from “Not at all” to “Nearly every day.” The total scores range from zero (0) to 27 with higher scores indicating more expressed depressive symptoms and a cut-off of ≥10 indicates moderate to severe depressive symptoms (35). The PHQ-9 is recognized as a sensitive measure for depression screening (39). Previous research indicated that the PHQ-9 is acceptable for use in major sociodemographic groups not only in clinical settings but also in the community (40). Scale was also previously used in students' research (41), and demonstrated potential value for the online screening programs (42). Internal reliability of the scale in the present sample was excellent with a Cronbach's alpha of 0.84.

The GAD-7 (43) is a seven item self-report instrument that is used to assess the severity of generalized anxiety disorder and anxiety symptoms. Each item asks the individual to rate the severity of his or her symptoms over the past 2 weeks using a four-point Likert scale with possible responses ranging from “Not at all” to “Nearly every day.” The total scores range from zero (0) to 21 with higher scores indicating more expressed anxiety symptoms. The GAD-7 was validated for the use in general (44) and students' populations (45, 46). It is recognized as a sensitive instrument for screening of anxiety disorders (47), with a cut-off of ≥10 indicating moderate to severe anxiety (43). Cronbach's alpha of the scale in the particular sample showed good internal reliability (α = 0.91).

The BIS-11 is a self-report scale, designed to assess personality and behavioral aspects of impulsivity (48). The scale consists of 30 items describing common impulsive or non-impulsive (for reverse scored items) behaviors and preferences. The items are scored on a four-point Likert type scale ranging from “Rarely/Never” to “Almost always/Always.” A higher total score indicates more expressed personality and behavioral aspects of impulsivity. The BIS-11 is the most widely cited instrument for the assessment of impulsiveness that was extensively used for impulsivity research in various populations and settings (49). A recent study of the psychometric properties of the BIS-11 in a Lithuanian adult sample demonstrated good construct validity, appropriate internal consistency, test-retest reliability, and prognostic value of BIS-11 in predicting addictive and delinquent behaviors such as smoking, alcohol consumption and law breaking (50). Cronbach's alpha of the scale in the current sample was 0.82.

### Statistical Analysis

Data were analyzed using IBM SPSS Statistics for Windows (version 20) and SPSS AMOS (version 20) (IBM Corp., Armonk, NY, USA). Before conducting the analysis, the data of the PIUQ-9, BIS-11, PHQ-9, GAD-7, and age were screened for missing values and normality. The normality of the distributions was assessed at the univariate and multivariate levels. Internal consistency was examined using corrected item-total correlations and Cronbach's alpha coefficient. Correlations were analyzed using Pearson's correlation coefficient and Spearman's r correlation coefficient.

Two-step cluster analysis was performed to group individuals into two clusters based on the questions reflecting habit changes due to COVID-19 pandemic: (a) the amount of time spent using internet and (b) purpose of the internet use. The One-way Multivariate Analysis of Variance (MANOVA) was conducted between two clusters (those with regular and those with increased frequency and changed purpose of internet use during COVID-19 pandemic) to investigate differences in the means of PIU, impulsivity, depressive and anxiety symptoms.

The structural equation model (SEM) was designed to test the mediating effect of impulsivity on the relationship between PIU, anxiety symptoms and depressive symptoms. The model fit was evaluated using the Chi-square test and the following indices: standardized root mean square residual (SRMR), goodness of fit index (GFI), comparative fit index (CFI), and root mean square error of approximation (RMSEA).

## Results

The cross-sectional study comprised 619 students (7.1% males, mean age 22 ± 3 years). The engagement rate of 45.8% was comparable with previously reported engagement rates in students' surveys (31). Majority of the students studied health and veterinary sciences (36.7%) and social sciences (30.2%). Detailed baseline characteristics of study population are presented in Table 1. In brief, 45.1% of included participants reported PIU, 38.1% of the participants had markedly expressed symptoms of anxiety, while 43.6% of students reported significant depressive symptoms. PIU correlated positively with anxiety (Pearson's *r* = 0.288, *p* < 0.001), depressive symptoms (Pearson's *r* = 0.356, *p* < 0.001), and impulsivity (Pearson *r* = 0.394, *p* < 0.001).

**Table 1:** Baseline characteristics of study participants.

**Characteristic**	**Total (*n* = 619)**
**Gender**, ***n*** **(%)**	
Male	44 (7.1%)
Female	575 (92.9%)
Age, mean ± SD	21,73 ± 2,571
**Field of study**, ***n*** **(%)**	
Mathematics and computer science	12 (1.9%)
Physical and biological sciences	61 (10%)
Engineering and technology	15 (2.4%)
Health and veterinary science	227 (36.7%)
Agricultural sciences	12 (1.9%)
Social sciences	187 (30.2%)
Humanities sciences	94 (15.2%)
Arts sciences	11 (1.8%)
The Problematic Internet Use Questionnaire score, mean ± SD	20,64 ± 6,346
The Patient Health Questionnaire, module for depressive symptoms score, mean ± SD	9,49 ± 5,497
The Generalized Anxiety Disorder Questionnaire score, mean ± SD	8,17 ± 5,394
The Barratt Impulsiveness Scale score, mean ± SD	42,42 ± 8,227
**Compared to the pre-pandemic coronavirus disease period, how did the time you spent using internet change? I spend …. time using internet**, ***n*** **(%)**	
A lot more time	217 (35.1%)
More time	253 (40.9%)
The same amount of time	132 (21.3%)
Less time	14 (2.3%)
A lot less time	3 (0.5%)
**How much did the coronavirus disease situation change your internet use habits? (When answering this question do not think about time spent using internet, but the nature and purpose of your internet use)**, ***n*** **(%)**	
Not at all	124 (20.0%)
A little	281 (45.4%)
Fairly	116 (18.7%)
Quite a lot	77 (12.4%)
A lot	21 (3.4%)

During the COVID-19 pandemic the amount of time spent using the internet (mean 4.7 ± 2.3 h) increased: 35.1 and 40.9% of students reported its substantial increase and moderate increase, respectively. The main purpose of the internet use was social networking (62.8%) and academic activities (24.1%). The increase in the amount of time spent on-line correlated positively with the lowered mood during COVID-19 pandemic (Spearman's rho = 0.215, *p* < 0.001) and depressive symptoms (Spearman's rho = 0.126, *p* = 0.002). The changes in the internet use habits correlated positively with the lowered mood during COVID-19 pandemic (Spearman rho = 0.182, *p* < 0.001).

Two-step cluster analysis included scores of time spent on-line and scores of internet use habit changes during the COVID-19 pandemic. The first cluster described respondents, who reported no changes in the amount of time spent online and habits of internet use during the COVID-19 pandemic. The second cluster included respondents, who reported increase in their amount of time spent on-line and changed habits in internet use during the pandemic. The ratio of the larger cluster size to smaller cluster was 1.23 with the average Silhouette measure of cohesion and separation of 0.6 showing good cluster quality.

Results of the MANOVA are shown in Table 2. The multivariate effect of the clusters on PIU, impulsivity, anxiety and depressive symptoms [Pillai's Trace = 0.022, *F*_(4.614)_ = 3.50, *p* = 0.008, Partial Eta Squared = 0.022]. During the COVID-19 pandemic anxiety and depressive symptoms were significantly higher in the second cluster of the frequent internet users (*p*'s < 0.05).

**Table 2:** One-way multivariate analysis of variance (MANOVA) for the differences in problematic internet use and mental distress symptoms.

**Indices**	**First Cluster** **(No change in internet use time and habits)** ***n* = 342**	**Second Cluster(Increasedinternet use time and habits*n* = 277)**	***F*_**(1,617)**_** ***F*-test statistics with the degrees of freedom df1 = 1 (for the between-groups estimate of variance) and df2 = 617 (for the within-groups estimate of variance)**	**Partial Eta Squared**	***P***
The Problematic Internet Use Questionnaire score, mean ± SD	20.0 ± 6.3	21.4 ± 6.3	7.52	0.012	0.006
The Patient Health Questionnaire, module for depressive symptoms score, mean ± SD	8.9 ± 2.3	10.3 ± 2.3	10.23	0.016	0.001
The Generalized Anxiety Disorder Questionnaire score, mean ± SD	7.8 ± 5.2	8.7 ± 5.6	4.62	0.007	0.032

Assessment of the univariate and multivariate normality was performed for the variables used in the SEM model. Multivariate outliers of the PIUQ-9, the BIS-11, the PHQ-9, the GAD-7 and age were removed using the Mahalanobis distance measure (critical value 20.51, Chi-squared test *p* = 0.001). Multivariate kurtosis and critical ratio were 2.96 and 4.40, implying multivariate normality in this sample.

**Figure 1:**
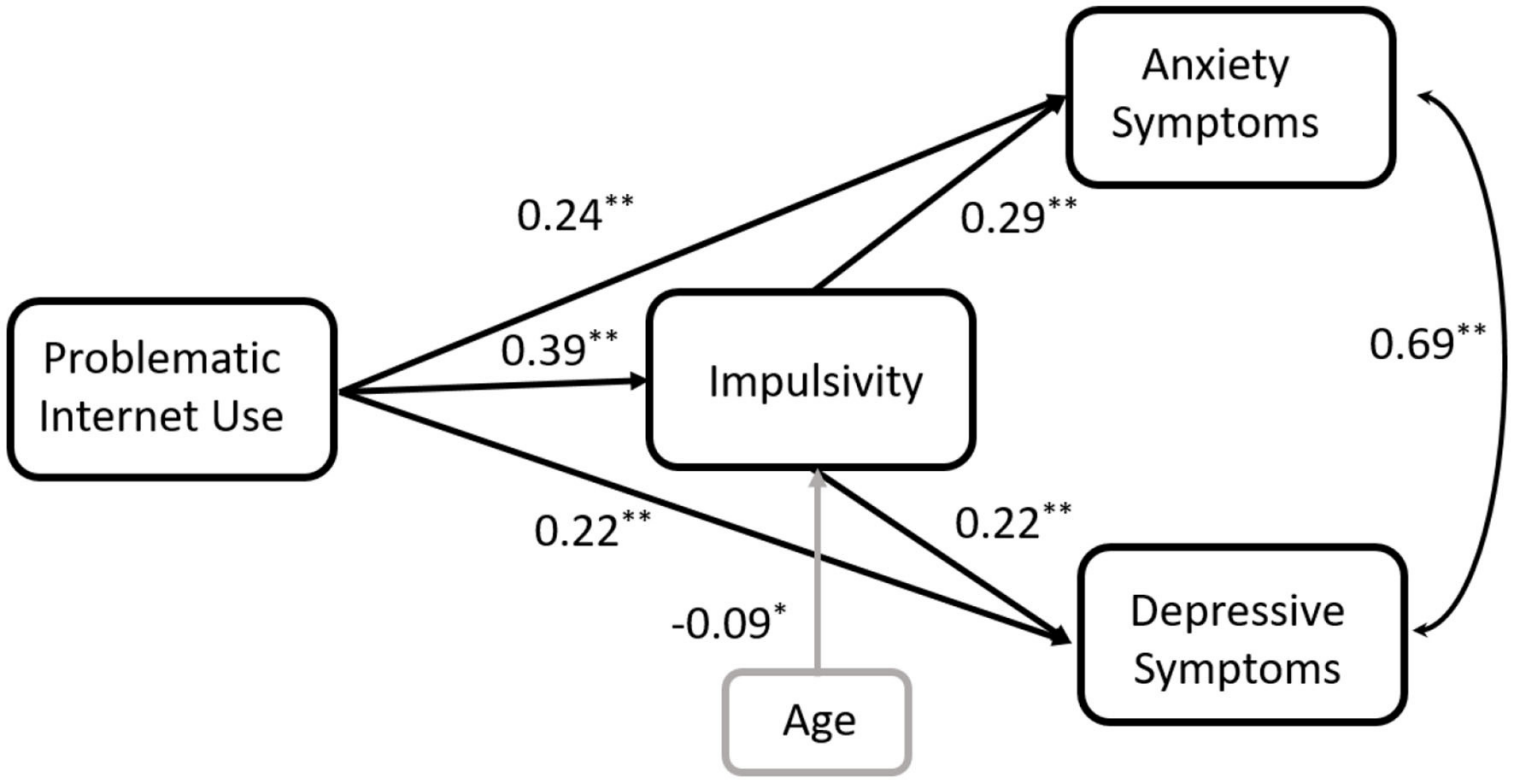
Structural equation model (SEM) testing of the mediational effect of impulsivity on the relationship between PIU, anxiety and depressive symptoms controlling for age. Chi-square value = 1.676, df = 3, *p* = 0.642, SRMR = 0.0104, GFI = 0.999, CFI = 1.00, RMSEA = 0.000. Path coefficients are standardized (*p < 0.05, **p < 0.001).

The results of the SEM analysis supported the hypothesized structural model (Chi-square value = 1.676, df = 3, *p* = 0.642, SRMR = 0.0104, GFI = 0.999, *CFI* = 1.00, *RMSEA* = 0.000). The model revealed the statistically significant effect of the PIUQ-9 on the GAD-7 (standardized direct path coefficient 0.200, 95% CI [0.124–0.292], *p* = 0.010; standardized indirect path coefficient 0.087, 95% CI [0.050;0.128], *p* = 0.010; standardized total effect 0.288, 95% CI [0.210–0.361], *p* = 0.010) and the statistically significant effect of the PIUQ-9 on the PHQ-9 (standardized direct path coefficient 0.240, 95% CI [0.155–0.320], *p* = 0.001; standardized indirect path coefficient 0.116, 95% CI [0.083;0.162], *p* = 0.010; standardized total effect 0.356, 95% CI [0.271–0.431], *p* = 0.010), mediated via impulsivity. The model accounted for 12.4% of the total amount of the GAD-7 variance and for 20.0% of the total amount of the PHQ-9 variance. Figure 1 shows the mediating role of impulsivity on the relationship between PIU, anxiety and depressive symptoms.

## Discussion

In the current study, we aimed to investigate the prevalence of PIU and mental distress symptoms during COVID-19 pandemic among university students in Lithuania. As the second aim, we tested the hypothesis that PIU affects anxiety and depressive symptoms through the mediating role of impulsivity.

Our study is among very few which analyzed the prevalence of PIU particularly in the population of young Lithuanian adults during the COVID-19 pandemic. Ninety five percent of individuals aged between 25 and 34 years reported using the internet daily, according to the National Statistic Department of Lithuania. However, most of the studies on the prevalence of PIU and associated risk factors focused on children and adolescents (51–54).

With regard to the first aim, we found that approximately 45% of students reported internet use behaviors and frequency that might be categorized as problematic, while around 38% and 44% reported significant symptoms of anxiety and depression during the COVID-19 pandemic, respectively. The prevalence of PIU was meta-analyzed in 2017, reporting 30.1% prevalence of PIU in medical students (10). Around one third of medical students also reported significant PIU in other recent studies by Anand et al. (11) and Shadzi et al. (55). The recent study, employing the same instrument for PIU with the same cut-off values, completed in Lithuanian students during Sept-Nov 2019 (37), found that 31.9% had symptoms of significant PIU. Thus, our study shows that the level of PIU is substantially higher during the COVID-19 pandemic than before this period. In addition, those subjects, who spent more time on the internet during COVID-19 pandemic, also had increased depressive and anxiety symptoms. This is an important finding for the further studies investigating effect of COVID-19 pandemic on individual psychological problems and well-being.

The present study also found positive correlations between PIU, depressive and anxiety symptoms as well as impulsivity. As hypothesized, both direct effect and indirect effect were significant, suggesting impulsivity as a mediator in the relationship between PIU and anxiety symptoms. Impulsivity also partially mediated the relationship between PIU and depressive symptoms, since both direct and indirect effects remained significant in the final SEM model. Our results were in line with Bisen and Deshpande (33) and Marzilli et al. (32) who reported significant links between PIU and depression, anxiety and impulsivity in students' populations. In the studies by Wang et al. (34) and Zhang (31) impulsivity was also a significant marker for PIU in the students. A higher score of internet addiction was also present in more depressive, more impulsive young adolescents in the study by Obeid et al. (56). Indirectly, our findings contributed to the current knowledge of high prevalence of PIU in depressive and anxiety disorders (57–60). The current research adds to the existing knowledge by examining the mediating role of impulsivity in the relationship between PIU and mental distress. However, due to a limited sample size, it was beyond our study scope to differentiate the impulsivity effect on depression and anxiety in the specific subgroups such as a group of students whose main purpose for using the internet is shopping or watching pornography or gambling. Recent studies show that these groups in particular might be prone to increased PIU symptoms (61–64).

Our study has several limitations worth noting. First, the study was based on the convenience sampling in university students in Lithuania, thus the generalizability of the results should be considered with caution. Second, the sample size precluded us from analyzing data from several perspectives including gender, purpose for the internet use and possible co-morbidity differences, as other studies show these to be the important characteristics to consider (61, 62, 64–66). The sample was mainly comprised by the female students and reflects the gender balance gap in the respective science specialities. It is important to note that the tendency of women participating in the surveys more often than men are documented in the earlier works as well (67, 68), possibly due to personality or gender role differences. However, the patterns of impulsive behavior (69) and PIU (70) has been observed to be distinct regarding the gender. Specifically, men tend to be more vulnerable to PIU symptoms (71) and have usually more severe symptoms (72), yet not difference among genders has also been reported (73). The interplay between impulsivity and gender is even more complex. Even though women tend to make impulsive choices more so than men, the eventual level of impulsivity depends on tasks and subject samples (69). Thus, the generalizability of our results to the men population is limited. Third, due to the cross-sectional nature of the study, we could not draw causal interpretations with regards to the relationship between PIU and mental distress, while considering the role of impulsivity. Thus, future longitudinal studies with larger and more diverse samples are highly encouraged. Despite the limitations, the current study was one of the first examining the prevalence of PIU among university students during COVID-19 pandemic as well as its interplay with mental distress and impulsivity.

## Conclusions

Almost half of the university students experienced significantly expressed PIU, anxiety or depression symptoms during the COVID-19 pandemic. Findings also suggest that the relationships between PIU, anxiety and depressive symptoms are partially mediated via impulsivity. These results underscore the importance of inclusion of impulsivity factor in the studies analyzing the longitudinal effect of PIU on mental distress during COVID-19 pandemic.

## Data Availability Statement

The study dataset is available upon request to Julius Burkauskas.

## Ethics Statement

The studies involving human participants were reviewed and approved by The Bioethics Center at Lithuanian University of Health Sciences. The participants provided their online consent to participate in this study.

## Author Contributions

JB, IG-B, and AP conceived and designed the study. LLD and GC designed the survey platform. JG-S, VL, DS, and RN were responsible for data collection and evaluation. Statistical analyses were performed by AS. JG-S prepared the manuscript together with AP. All authors provided critical revision to its further development, read, and approved the final manuscript.

## Conflict of Interest

JG-S serves as a consultant at FACITtrans. In the past several years JB has been serving as a consultant to Cogstate, Ltd. The remaining authors declare that the research was conducted in the absence of any commercial or financial relationships that could be construed as a potential conflict of interest.
